# 

**Published:** 1895-07-13

**Authors:** 


**Notices of Births, Deaths, and Marriages** are inserted in this column at a charge of 2s. 6d. for an announcement not exceeding four lines.

**Notice to Advertisers and Subscribers.**

All Advertisements, Orders for Copies of The Hospital and Business Communications should be addressed to **The Manager, NOT TO THE EDITOR**, The Hospital, 428, Strand, London, W.C.

The Cost of Subscription, post free, is as follows:—Per week, 2½d.; per quarter, 2s. 8d.; per half-year, 5s. 3d.; per annum, 10s. 6d. Foreign:—Per Annum, 15s. 2d.; payable, in all cases, in advance.

### NOTICE TO CORRESPONDENTS.

All MS., Letters, Books for Review, and other matters intended for the Editor should be addressed The Editor, The Lodge, Porchester Square, London, W.

The Editor cannot undertake to return rejected MS., even when accompanied by stamped directed envelope.

**"Burdett's Hospital Annual," 1895,**
Now Ready.

Sixth year of publication. 950 pp. Scarlet cloth, gilt lettered. Price 5s. A most complete guide to British, Colonial, Indian, and American Hospitals, Dispensaries, Nursing and Convalescent Institutions, and Asylums. A few copies of the "Annual" for 1894 may still be obtained on application to the Publishers, The Scientific Press (Limited), 428, Strand, London, W.C.

**NOTICE.—Vol. XVII. of "The Hospital" (October, 1894, to April, 1895), in handsome cloth binding, is on sale at the Office of this Journal. Price 6s. Cases for binding the Numbers contained in this Volume, 1s. 6d. Index to Vol. XVII., 2½d., post free.**

**The Monthly Part for July is now ready, price 6d.; by post, 8d.**

### Scraps and Gleanings.

An infectious diseases hospital is being built for the Corporation of Heywood at Bird i' th' Hand, Birtle.

Recent donations to the Princess May Ward at the Royal Richmond Hospital amount to fifty-seven guineas.

One of the many American medical colleges of St. Louis has been sold to three doctors, who intend organising a new faculty.

The Aberdeenshire Football Association has given £50 to the Royal Aberdeen Infirmary and £10 to the Convalescent Hospital.

The Fazakerley Cottage Homes Committee (West Derby) have decided to erect a corrugated iron hospital for the accommodation of infectious cases.

The Zoological Society of Bristol have offered to place their grounds at the disposal of the Bristol Hospital Saturday Committee, should they be needed for the purpose.

Out of a total of £2,600 received at Devon and Exeter Hospital, according to the present year's report, no less than £2,554 is included under the head of "legacies."

The successful performance by the Marlowe Dramatic Club of "Arrah-na-Pogue" resulted in a contribution of £80 towards the funds of the Great Northern Central Hospital.

The Mile End Guardians have, it is stated, passed the plans for cottage homes for pauper children at North Weald, Essex. The estimated cost is from £40,000 to £50,000.

During last year over £1[illegible]0 was expended by the committee of the General Hospital, Jersey, for the maintenance of the "Overdale" Hut, though two cases alone applied for treatment.

An international congress of applied chemistry will be held in Paris next year. A preliminary programme of the sections has been drawn up for consideration. They are ten in number, and cover the entire field of industrial chemistry.

The experiment of an electrotherapic service has been started at the Lariboisière Hospital at Paris, under the recommendation of the principal officials of that establishment. Dr. Plicqué has been appointed director of the service.

It is curious to read of a definite geographical migration. Cape Canaveral, on the coast of Carolina, is presenting a distinct feature in this particular. When first known it was forty miles further north, and this southward migration is still going on, but at a creeping pace.

The annual procession and open-air concert in aid of local charities has just taken place at Wrath. The institutions which the demonstration is intended to benefit are the Sheffield General Infirmary, the Leeds General Infirmary, and the Montague Cottage Hospital at Mexbrough.

A meeting of the Wroxall Parish Council was held last week to consider a proposal of the Ventnor District Council to include the parish of Wroxall in a new hospital district to be created under the provision of the Hospital Act. This proposal has not, so far, received much encouragement.

For some years past the question of the enormous increase in the number of out-patients of the Hertford General Infirmary has received the serious consideration of the Board. It has now been found necessary to enlarge the present building to keep pace with the demands thus made upon its accommodation.

There is still room for more generous treatment of the claims of the Dundee Infirmary. A heavy increase in its work has been attended by a steady decrease in its funds, and the excess of expenditure over income last year was £1,540. This failure of support is not attributable to the working classes, who contribute to the best of their ability.

Since the last report of the Glasgow Public Dispensary has been issued a house of four rooms and kitchen immediately above the dispensary have been leased with a view of forming it into an hospital to be used in connection with it. Part of this is for a ward and the nurse in charge of the same. During the period under review the report of the dispensary shows that 3,706 consultations have been held.

A convalescent home is about to be added to the Aberdeen Royal Infirmary. The directors are, therefore, soliciting further financial aid towards the claims of this institution. The trustees of Mr. John C. Chalmers have allocated the munificent sum of £5,000 towards this new convalescent hospital, £2,000 of the sum to assist in providing ground and new buildings, and £3,000 to be kept intact as capital.

In the course of the year 230 patients have been received as in-patients at the Drumcondra Hospital, Dublin, being the highest number received at any period in the existence of the hospital. Although the account sheet for the year shows a small credit balance to exist at the bank, yet the finances of the hospital are not in a wholly satisfactory condition, and further funds are earnestly asked from the charitable public.

### The Student of Medicine.

### Appointments.

P. E. Adams, M.R.C.S Eng., L.R.C.P.Lond., appointed Junior House Physician to the North-Eastern Hospital for Children, Hackney Road. W. A. Bond, M.A., M.D., B.C.Cantab., D.P.H.Cantab., M.R.C.P.Lond., M.O.H. for the St. Olave District Board, Southwark, appointed M.O.H. to the Holborn District Board. W. B. Brodie, M.B., C.M Glasg., appointed Assistant House Surgeon and Dispenser at the Worcester Infirmary. E. Carver, M.A., M.D., appointed Consulting Surgeon to Addenbrooke's Hospital, Cambridge, on retirement from the Surgeoncy. A. O. Davies, L.R.C.P., L.R.C.S.Edin., appointed Medical Officer of Health for the Machynlleth Union District. E. H. Douty, M.A., M.B., B.C., M.R.C.S., L.R.C.P., appointed Assistant Surgeon to Addenbrooke's Hospital, Cambridge. A. Fausset, M.B., appointed Medical Officer for the No. 2 District of the St. George's Hanover Square Union. W. Foster, B.A. Camb., M.B., D.P.H., appointed Medical Officer of Health to the Shipley District Council. J. Griffiths, M.A., M.D., F.R.C.S., appointed Surgeon to Addenbrooke's Hospital, Cambridge. J. F. Haswell, M.B., C.M.Edin., M.R.C.S, appointed Medical Officer of Health to the Penrith Rural District Council. A. Howie, M.B., C.M.Glasg., appointed Medical Officer for the Alberbury District of the Atcham Union. F. Hunton, M.D.Durham, appointed Medical Officer for the Stockton District and Workhouse of the Stockton Union. H. T. Jones, L.R.C.P., M.R.C.S., appointed Medical Officer of Health to the Pembroke Rural District Council. W. Muir, M.B., C.M.Glasg., appointed Medical Officer and Public Vaccinator to the Parish of Cramond, Midlothian. H. N. Pelly, L.R.C.P., L.R.C.S.Edin., appointed Medical Officer for the Second District of the Winslow Union. Mr. G. Pickering appointed Medical Officer and Public Vaccinator for the Ninth District of the Hexham Union. J. Priestley

**THE STUDENT OF MEDICINE**—*continued.*

B.A.Lond., M.D Edin., appointed Medical Officer of Health for Lambeth. M. H. Quarry, M B R.U.I., B.Ch., appointed Medical Officer of the Lambeth Infirmary and Workhouse. T. Smith, M.R.C.S.Eng., L.R.C P.Lond., appointed Medical Officer and Public Vaccinator for the Brampford Speke District of the St. Thomas's Union. E. Semerset, L.R.C P.Lond., M.R.C.S. Eng., appointed Medical Officer of Health for the Oundle Union District. W. H. B. Stoddart, M.B., B.S., appointed Senior House Physician at the National Hospital for the Paralysed and Epileptic, Queen Square, Bloomsbury. G. Turner, M B. Camb., D.H.P., L R C.P.Lond., appointed Medical Officer of Health for the Cape Colony. Mr. Vallance, appointed Assistant Medical Officer to the Chelsea Union. R. Young, B.A.Durh , M.D., M.R.C.S.Eng., appointed Medical Officer of Health for the Royton Urban District.

### VACANCIES.

The following vacancies are announced this week, the amount of salary in each case being given after the name of the institution:

#### Resident Medical Officers.

Birmingham General Dispensary.—Resident Surgeon; doubly qualified. Salary £150 per annum, with an allowance of £30 per annum, for cab hire, furnished rooms, fire, lights, and attendance. Applications to Alex. Forrest, Secretary, by July 15th.

Cancer Hospital Free), Fulham Road, S.W.—House Surgeon. Appointment for six months. Salary at the rate of £50 per annum, with board and residence. Applications to the Secretary by July 15th.

City of London Hospital for Diseases of the Chest, Victoria Park, E.—Resident Medical Officer; doubly qualified. Salary £100 per annum, with board, &c Applications to the Secretary by July 19th.

Coventry and Warwickshire Hospital.—Resident Assistant House Surgeon. Appointment for six months. Salary £15, board (exclusive of beer, wines, and spirits), residence, washing, and attendance. Applications to the Secretary by July 20th.

Hospital for Consumption and Diseases of the Chest, Brompton.—Resident Assistant Physician. Applications to the Secretary by July 18th.

Flintshire Dispensary.—Resident House Surgeon. Salary £120 a year, with furnished house, rent and taxes free, also coal, light, water, and cleaning, or in lieu thereof a sum of £20 per annum. A knowledge of Welsh desirable. Applications to Thos. Thomas, Secretary, Board Room, Bagillt Street, Holywell, N. Wales, by July 17th.

Newport and Monmouthshire Infirmary, Newport. Mon.—House Surgeon; doubly qualified. Salary £100 per annum, with board and residence. (No stimulants provided.) Applications to the Secretary by July 13th.

National Sanatorium for Consumption and Diseases of the Chest, Bournemouth.—Resident Medical Officer. Salary £80 per annum, with board, lodging, and washing. Applications to the Secretary by July 25th.

Royal Berks Hospital, Reading.—Third or Assistant Medical Officer. Board, lodging, and washing provided, but no salary. Appointment for six months. Applications to the Secretary by July 13th.

Royal Victoria Hospital, Bournemouth.—House Surgeon and Secretary. Salary £100 per annum, with board. Appointment for two years. Applications to the Chairman of the Committee by July 17th.

Sheffield General Infirmary.—House Surgeon and Senior Assistant House Surgeon, doubly qualified. Salary for the former, £120 per annum, with a prospective advance of £10 per year for the second and third years; and for the latter £80 per annum, with board, lodging, and washing. Applications to the "Medical Staff of the Sheffield General Infirmary, to the care of the Secretary," by July 13th. The election will take place on July 26th.

Suffolk General Hospital, Bury St. Edmunds.—Horse Surgeon. Salary £100 per annum, with board, lodging, and washing. Applications to Henry Bonner, Secretary, by July 15th.

#### Non-Resident Medical Officers.

Bradford Infirmary and Dispensary.—Honorary Physician. Applications to the Secretary, by July 22nd.

British Institute of Preventive Medicine.—Assistant Bacteriologist in the Anti-toxin Department. Salary £150 a year. Applications to the Director, by July 22nd.

Queen's Jubilee Hospital, Earl's Court.—Surgeon. Applications to the Secretary by July 14th.

University of Glasgow.—Two Examiners for Degrees in Medicine to Examine in Chemistry and Materia Medica respectively. Applications to Alan E. Clapperton, Secretary of the Glasgow University Court, 91, West Regent Street, Glasgow, by July 25th.

### Medical and Administrative Appointments.

GREAT NORTHERN CENTRAL HOSPITAL,

